# COVID-19: Reflections on the Crisis, Transformation, and Interactive Processes Under Development

**DOI:** 10.1007/s43076-020-00061-z

**Published:** 2020-12-29

**Authors:** Gabriella Garcia Moura, Célia Regina Rangel Nascimento, Juliene Madureira Ferreira

**Affiliations:** 1 Department of Social Psychology and Development, Federal University of Espírito Santo, Vitória, ES Brazil; 2 Department of Psychology, Federal University of Espírito Santo, Vitória, ES Brazil; 3 Faculty of Education and Culture, Tampere University, Åkerlunkinkatu, 5. Office 509, FI-33214 Tampere, Finland

**Keywords:** Pandemics, Crises, Changes, Human development, Social interactions

## Abstract

With a global extent, the pandemic of the new coronavirus and the resulting measures to contain the contagion imposed immediate changes in the routine of people and societies. In view of this historical event, the first part of this theoretical study discussed its relationship with the concept of crisis, while circumscribing human development processes, mobilizing reorganizations in life trajectories. In the second part, the intensification of the use of digital tools to support communication during social isolation was highlighted, particularly reflecting on new interactive arrangements and inter-corporeal experiences. The paper reflects on the proximal processes in the new interactive and contextual configurations through the bioecological theory of human development and, based on concepts of the enactive theory, discusses possible implications of the new perceptual fields and the production of meanings with the repositioning of the body and new modes of engagement. The study highlights that the changes, events, relationships, and effects that we are experiencing (trans)form our forms of sociability and bases of psyche.

## Introduction

In January 2020, the World Health Organization (WHO) declared that the COVID-19 epidemic constituted a Public Health Emergency of International Concern (WHO 2020a). The disease, identified in December 2019, had already reached different places in the world, imposing safety measures against contagion and raising the alertness of health systems in several countries. In addition to rapid contagion, there was a risk of death among some of the most susceptible groups, which resulted in the need to instruct the population to maintain social isolation (i.e., avoidance of physical contact, locomotion, and agglomerations, in addition to the closure of schools, shopping centers, and borders) to prevent the spread of the disease on a large scale and the overload of health systems with the treatment of more serious cases (Oliveira et al. 2020). The restrictive measures varied according to the reality of each country, but they imposed rapid and immediate changes in the daily lives of people and societies.

With a worldwide impact, the spread of the new coronavirus (SARS-CoV-2) created a scenario of many uncertainties. For example, it is uncertain how to contain, control, treat, or cope with the disease, and predict the duration of the pandemic and its effects, resulting in countless challenges and overloading of personal and social resources. It is certainly a stressful event, with impacts already visible in the daily lives of individuals and institutions, imposing the need to reorganize social life, the educational system and routines, work environments and work routines, and leisure activities, in addition to the possibility of experiencing the disease and the losses resulting from it (Brooks et al. 2020; Shojaei and Masoumi 2020). It is, therefore, an event that mobilizes personal and collective crises.

In front of this historical event, the present theoretical study presents reflections on the transformative processes experienced with the COVID-19 pandemic organized in two central topics. In the first topic are addressed theoretical concepts that put the pandemic in perspective as a critical (or “crisis”) event, mobilizing and circumscribing changes, vulnerabilities, reorganizations, and new life structures (Levinson 1978; Sheehy 1974; Zittoun 2007). For such reflection, the study focuses on the concept of transitions and crises and their repercussions for human development processes, that is, on understanding the processes of change from life events and their influences (Baltes et al. 1999; Neri 2012). The developing person was considered as part of a network of relationships constituted *in* and a constituent *of* physical, social, and culturally organized environments; and the importance of people’s interactions with other people, symbols/signs, times, spaces, and social positions/papers is taken into account at all times (Rossetti-Ferreira et al. 2007; Zittoun 2007).

In agreement, in the second topic, some of the changes and transformations in interpersonal interactions produced by social isolation are analyzed, considering social interactions as arena and engine of development processes (Rossetti-Ferreira et al. 2007). For such analysis, the focus was shed on new interactive arrangements and embodied experiences, particularly with the intensification of the use of virtual communication tools. This topic is organized in two subtopics. In the first, reflections on the proximal processes in the new interactive configurations and altered ecological contexts are addressed from the systemic perspective of the bioecological theory of human development (Bronfenbrenner and Morris 2006). In the second, the different perceptual frames and meaning-making are discussed considering the repositioning of the body and new modes of engagement in social interactions through the perspectives of embodiment in the theory of enaction (Varela et al. 2017).

The theoretical perspectives that are discussed in this study are aligned in terms of the understanding that life trajectories, human development, and cognition cannot be understood disconnected to their socio-cultural context, apart from the social interactions that circumscribe how a person experiences, thinks, dreams, imagines, and understands the world. Therefore, it was highlighted how critical events, changes, relationships, and affections that we are experiencing (trans)form forms of sociability and bases of our psychism.

## Transitions and (Un)predictable Crises: Changes and Transformations with the COVID-19 Pandemic

Life trajectories and their intrinsic development processes are not only successive periods of stability but also involve ruptures and transitions (Rossetti-Ferreira et al. 2007). As defined by Zittoun (2007), a rupture can result from an important change in the cultural context (e.g., a war, a pandemic), a direct change in a person’s spheres of experience (e.g., a change of workplace, a new arrangement of the children’s routines), changes in the relationships that a person establishes with others and objects (e.g., intensified the use of virtual communication tools, family coexistence in the domestic environment), and from changes in the persons themselves (e.g., learning new skills, changes in emotional state). As it is possible to notice, within the context of the pandemic, all these different ruptures can be identified.

In all these cases, ruptures demarcate transition periods defined based on their structure and psychosocial dynamics (Zittoun 2007). In terms of structure, “transitions in the course of life designate processes of adjustment to new life circumstances. Usually, transitions follow ruptures - modifications of what is taken-for-granted in a person’s life” (Zittoun 2007, p. 03). In terms of dynamics, transitions involve three types of interdependent processes. First, changes in the spheres of experience of the person, in social, material, and symbolic terms. The ruptures impose a repositioning of the person in his social and symbolic field. New goals, orientations, (im)possibilities of action, losses, and gains are created. Such repositioning also implies identity changes. Second, these relocations/replacements mobilize new social, cognitive, and specialized forms of knowledge and skills (e.g., learning new tasks developing new everyday activities). And, thirdly, in these reallocations and encounters with other people and learning, the person is led to engage in new processes of meaning, continuously building meanings and attributing meaning to the phenomena around him/her, to the events he/she experiences (Zittoun 2007).

Events such as those resulting from the pandemic, which has an impact on the lives of individuals, leading to an imbalance and the need for reorganization in the search for new stability, also have been studied in Developmental Psychology from the concept of “life event” (Neri 2012). A life event is defined from different perspectives that can focus on the life event as a stress-generating event (Miloya et al. 2018; Woyciekoski et al. 2014) or as an event that changes the individual’s status development (Bleidorn et al. 2018). According to Woyciekoski et al. (2014), a life event refers to a daily event that can be psychological or physical and which can mobilize changes in the person’s routine in the personal or social domains. Life events can be considered to influence human development, insofar as they impose the need for adjustments and changes to cope with them, which may result in cognitive, behavioral, or affective changes (Bleidorn et al. 2018), such as the establishment of new relationships, skills, and abilities or new understandings of the environment in which people live. Life events can be expected due to biological development processes or social and cultural expectations, but they can also be unexpected. They can be evaluated negatively or positively or as events that involve both positive and negative experiences. It is an evaluation that occurs according to how it impacts the individual’s life, how it is perceived, and the personal and social resources to deal with the event (Neri 2012).

Considering the plasticity of developmental processes throughout life (*lifespan perspective*), Baltes et al. (1999) describe three types of events that influence and make up the ontogenetic architecture: *age-graded events*, which comprise the predictable changes related to maturation, organic growth, aging, and socialization processes typical of each age group (events—biological or social—with high frequency in each age group); *history-graded normative events*, which correspond to macro-structural events experienced at the same time by groups of people at a given historical moment, in a given society; and *non-normative events*, relating to unpredictable events that do not affect all individuals in an age group, thus not depending on ontogeny or historical time. The current historical and social context of the new coronavirus pandemic puts the characteristics of both historical and non-normative events in close articulation. In other words, the covid-19 pandemic represents a global health crisis with profound economic and social repercussions never before experienced in the contemporary globalized scenario, already being considered a historical event, whose effects are imposed at the same time on several generations. And like the non-normative events described by Baltes et al. (1999), the pandemic is experienced in a varied way depending on the different socioeconomic, age, ethnic, and gender groups. Its effects may vary according to the meanings of the event for each person, the roles and social positions he/she occupies, and his/her conditions and coping strategies.

Therefore, changes faced throughout life can be experienced as events that mobilize crises and conflicts, demarcating critical transition periods. Developmental Psychology is interested precisely in the processes of constitution of individuals in the flow of these events, with a special focus on (resulting) current changes and transformations in their psychosocial, affective, cognitive, and communicative aspects, as well as in aspects that remain stable throughout the course of life (Barros and Coutinho 2020; Rossetti-Ferreira et al. 2007). In fact, many crises are predictable and expected in the course of life (described as normative events), for example, the birth of a younger brother who causes jealousy, a change of school, the start of puberty, the beginning of a marriage, or its end, with divorce, entering the labor market, and several other situations that represent turning points, periods of shock. However, unexpected events can lead to unpredictable crises.

### Unpredictable Crises and the Pandemic

A significant difference between predictable and unexpected life crises resulting from the blow represented by a fatality or accident lies on the temporal dimension. That is, in expected circumstances, there is time to adjust; coping possibilities are revealed and developed over time and may even be preceded. However, “when [crises] are suddenly thrown at us, we are unable to accept and deal with them immediately. They arrive with too much force and too quickly” (Sheehy 1974, p. 07), as the global crisis experienced with the spread of Covid-19, which has had repercussions in crises in the macro and micro-systemic scope.

With such strength and speed, the pandemic has abruptly driven several countries to the collapse of their traditional health systems, social, and economic functioning (Villarroel 2020). Recent studies have reported impacts of the pandemic on the mental health of the general population, like emotional destabilization and exacerbated reactions of fear in the face of uncertainties, acute stress, anguish, fatigue, anxiety, episodes of panic, sadness, hopelessness, disorganized sleep, and avoidance of social contact, especially in health workers (Bezerra et al. 2020; Brooks et al. 2020; Schmidt et al. 2020; Shojaei and Masoumi 2020). The difficulty of coping associated with the adoption of inappropriate or poorly adaptive strategies (such as increased consumption of alcoholic beverages and other drugs) has also been observed, and even the denial of risk and illness, minimizing the severity of COVID-19 and ultimately leading to exposure to unnecessary risks (Schmidt et al. 2020; Villarroel 2020). In addition, family, marital, and parental conflicts have become more strained with the confinement of families, leveraging rates of aggression and domestic violence (Cluver et al. 2020). Finally, the occurrence of xenophobia, discrimination, and stigmatization has been intensified (Villarroel 2020). Similar effects have been observed in studies resulting from other epidemics, health emergencies, and natural disasters, which have also triggered significant emotional destabilization in the general population (WHO 2020b).

It is observed that, in the case of individuals and families who already lived other adversities previous to this event, these adversities were likely aggravated by the pandemic. The accumulation of tensions and the absence of support from a protective social and institutional context can overload people and families even more, configuring a situation of vulnerability. Bezerra et al. (2020) investigated the changes caused by social isolation in Brazil and observed that the people most affected economically, in the sense of not being able to (keep) have the family income during isolation, were precisely those who before the pandemic belonged to the groups of people with the smallest income. There was also a correlation between the impact on income and family stress; participants with a higher number of people in the household and with lower quality of housing mentioned family stress as the greatest impact of isolation. Thus, although social isolation is a protective measure to avoid contagion by coronavirus, it can also act as a personal and family risk factor depending on the duration and the effect on the living conditions and relational dynamics.

It can be seen, therefore, that periods of crisis can also represent periods of greater vulnerability (Sheehy 1974). Although there is no single definition for the term, the concept of vulnerability has been used in different areas of knowledge to address situations that involve risk and assess its impact and the possibilities for dealing with it (Wisner 2016). In the health field, the term vulnerability was highlighted with the HIV/AIDS epidemic from reflections on the susceptibility of some groups and the relationship between care, prevention, and human rights. The articulation between individual, social, and institutional dimensions was emphasized in the assessment of the conditions of vulnerability and coping of groups and people. At the individual level, it is considered important to assess the degree of exposure to the risk factor, knowledge and access to information, conduct and practices aimed at protection, as well as the subjective assessment of the own condition. In the social sphere, contextual aspects and aspects related to social inclusion, such as economic situation, gender, ethnicity, social exclusion, and other social conditions that affect the ability to access resources and support, are relevant factors. In the institutional or programmatic scope, the availability of services and the conditions to access them stand out, in addition to the policies and programs that make it possible to monitor and develop actions to promote well-being and care (Garcia and de Souza 2010; Oviedo and Czeresnia 2015). Thus, “Vulnerabilities are defined in the relationship with the other, whether it is a person or social equipment” (Garcia and de Souza 2010, p.11), implying both the possibility that damage will occur in the face of a risk situation and the conditions for coping with this situation.

In the case of COVID-19, its high rate of transmissibility; the absence of vaccines, tests, and of the capacity of health services to serve all cases (Oliveira et al. 2020); and the constant changes and different approaches of the political and administrative sectors to the pandemic, in addition to the need for remoteness and social isolation, implying less access to support networks, make the feeling of vulnerability widespread.

### Crisis and Coping Processes

Analyzing the health crisis imposed by COVID-19 as a turning point, as a constitutive passage, mobilizing new adaptations and opportunities, it is valid to reflect on the possible changes not only in the sense of illness but also coping and reorganization. Interested in understanding the crises of adult life, Sheehy (1974) states that the Greek word *krisis* (whose meaning relates to “decision,” “decisive moment”) is pejoratively interpreted in Western culture, which attributes a sense of personal failure, weakness, and inability to resist pressing external facts. For this reason, the author chooses not to use the term “crisis” but “passage,” understanding that this word would better reflect the quality of critical transition between periods, denoting the interaction of stable periods and the critical turning points.

In a similar vein, Levinson (1978) explains psychosocial development from a sequence of alternating periods of stability and transition, in which life structures are constructed and modified. The transition period necessarily involves processes of adaptation, urgent questioning, exploration of the possibilities for change, and evaluation of the current structure. These transition periods are not determined by chronology; they are rather determined by their potential to allow an assessment of what it has been lived, of the past, and of new choices about what to bring to the next stable life structures (Coutinho 2010). Along the same lines, Sá et al. (2008) recall that the Chinese character of crisis is represented by two figures: one meaning “danger” and the other meaning “opportunity,” that is, “point of change.”

In this sense, analyzing the various lessons that the pandemic has already made possible to learn and the changes that can be established from this event and resulting experiences, Manoukian and Manoukian (2020) highlight some points, namely, the profound changes in the use of space and time; the discovery of the relational micro context; the difference between physical distance and social distance; the potential for civic mobilization, with several initiatives of solidarity and empathy; the importance of prevention; and, mainly, the tight relationship between health and social aspects,. The authors reflect on how the pandemic highlights our networks of belonging, by imposing interpersonal distance. It expands the perception of who are the people with whom we establish significant interactions, with whom we share interests, trajectories, passions, and joint activities. In this sense, the pandemic has taught “how much our well-being is built in the micro contexts in which we live” (p. 45). Another relevant change refers to the center of gravity of medicine, which has moved from hospitals to territories. For the authors, in recent years, the privileged investment in hospitals, centers of excellence, and specializations has emptied medicine of territory, prevention, and proximity. Strengthening the “myth of technique” as well as individualistic approaches, isolated from the context in which one lives. With the pandemic, the role of specialists is needed to be shared with individuals, who had to develop their basic health skills and knowledge about health and illness. If the thought “if I get sick, they will cure me” was prevalent until then, the pandemic showed that “it was necessary to have avoided the pulmonary infection caused by the virus, so that patients did not arrive at the hospital in a critical condition” (p. 44). Furthermore, the authors evaluate that the pandemic reinforces that the persons’ cure is also related to the reception and emotional support of a relational network that helps them to go through difficulties, with the occupation of time with purpose and meaning, in addition to the possibility of learning to deal with their suffering in order to get stronger (Manoukian and Manoukian 2020).

Considering the importance of the individual’s perception of the traumatic event, Sá et al. (2008) describe different stages in experiencing and coping with a crisis, ranging from emotional disorganization to a stage of elaboration, in which it is possible to face the experience and express and identify the feelings, allowing to integrate the event in the person’s life, expanding problem-solving skills, and reframing them. In this sense, the impact of the COVID-19 pandemic will also depend on how people, communities, or family members deal with the situation and on key factors that can stimulate personal, community, and family strengthening and growth in the face of crises and adversities, favoring resilience processes.

According to Grotberg (2005), a resilient behavior “requires preparing for, living and learning from adverse experiences” (p. 17). In general, it is considered that in the human development process, resilience is related to the ability to overcome adverse situations that cause stress, and which lead to the transformation and strengthening of individuals and groups (Melillo et al. 2005; Grotberg 2005). Studies on the topic have shown that resilience is a potentiality, implying the relationships established throughout life, developing in relation to internal and environmental protective factors and in the dynamic interaction between individual characteristics and the contexts in which the person is inserted (Walsh 2016). Therefore, it has been highlighted that resilience is, above all, a potential that presupposes the interaction between people (Melillo et al. 2005; Walsh 2016).

In an approach that considers resilience processes, while considering the suffering and negative consequences that may arise from the adverse event, there is also an optimistic perspective of the changes brought about by a crisis, assuming that “A crisis can be a wake-up call for important issues. It can become an opportunity to reevaluate priorities and goals in life, encouraging greater investment in meaningful relationships” (Walsh 2016, p. 401).

In view of all these reflections that focus on the “critical” aspect of the pandemic as a constitutive and transforming event in life courses, it is evident how much changes are part of the human development process and happen in different domains (physical, social, psychological). Losses and gains are part of this process (Lally and Valentine-French 2019) that can be triggered by different situations and challenges imposed by life. At this time, and like other transitions that we experience throughout life, COVID-19 and the resulting measures of social isolation are already important influences on human development trajectories, circumscribing different meanings in the courses of life. Like other periods of transition and changes, losses, as well as new competences, skills, behaviors, tools, and experiences are being established, from the macro-social and political-economic to the personal (psychomotor, cognitive, affective) spheres. Such changes occur interdependently with the historical context and the physical, social, political, geographical, and symbolic environments (Rossetti-Ferreira et al. 2007). Therefore, although the pandemic is transitory, the events, relationships, and attachments that we are experiencing are embodied and (trans)form the basis of our psyche and our forms of sociability.

It can be seen that there are several theoretical perspectives dedicated to the analysis of the changes and (trans) formations that constitute life trajectories, whether during periods of crisis and transitions or of stability. Within this diversity, there are few doubts and disagreements regarding the importance of the interactions of the organism with the environment in development processes. Particularly, it is understood the constitutive processes anchored in and through the actions and interactions established by individuals with other people in physical, social, and culturally organized environments. The developing person is understood as part of systems of relationships (of networks of interactions and relationships) and, therefore, the analysis of the processes of change considers the relationships between the individual, people, and the phenomena of his surroundings (Rossetti-Ferreira et al. 2007). Therefore, considering social interactions and relationships as an arena and engine of development processes (Rossetti-Ferreira et al. 2007), the question is how does the pandemic bring new circumscribers to our interactive constitutive experiences?

## Transformations of Interactive Processes with the COVID-19 Pandemic

The human being, as a biologically social being, is constituted in the interactions with others, in the process of understanding and relating with the other, the world, and the social-historical-cultural productions (Rossetti-Ferreira et al. 2007). The development of psychological studies on social interaction led to the understanding of interaction as a process and triggered research to look for the quality of interactions and their meanings for the subjects involved considering the bidirectionality of interpersonal social relations. From this perspective, studies in different contexts have historically been conducted by different authors and theoretical perspectives (Aranha 1993).

### Transformations in the Contexts of Interactive Processes

The interactions assume a prominent place in the perspective of the bioecological theory of human development (Bronfenbrenner and Morris 2006). Since the beginning of the theory’s elaboration, the understanding that interactions in different ecological contexts are the focus of studies on human development is evident (Merçon-Vargas et al. 2020). Throughout the maturation of the theory, interactions started to have greater emphasis, particularly with the discussion about proximal processes, referring to the lasting and significant forms of interactions in the immediate environment, of coexistence. Therefore, in this perspective, the environments in which proximal processes actually occur are the surrounding environments, that is, in microsystems, defined as:

A microsystem is a pattern of activities, social roles, and interpersonal relations experienced by the developing person in a given face-to-face setting with particular physical, social, and symbolic features that invite, permit, or inhibit, engagement in sustained, progressively more complex interaction with, and activity in, the immediate environment. (Bronfenbrenner and Morris 2006, p.814)

Proximal processes can be established between people and also with symbols and objects. When they occur between people, in face-to-face interactions, they imply mutual engagement in a pattern of activities, involving reciprocity, attention and affection, sustained with frequency and continuity, and with progressive levels of complexity over time. When the person’s interaction occurs with symbols or objects, they stimulate attention, exploration, manipulation, elaboration, and imagination (Bronfenbrenner and Morris 2006; Merçon-Vargas et al. 2020). The potential of proximal processes to promote development is considered to vary depending on the characteristics of people, the characteristics of the environment in which proximal processes take place, and the changes and continuities that occur throughout the life cycle and the historical period in which the person lives. Proximal processes are also influenced by different contexts, as people interact in different environments throughout life and bring their experiences, cultures, and values ​​to relational processes (Bronfenbrenner and Morris 2006).

During the COVID-19 pandemic, access to different social contexts as well as forms of engagement and the duration of interactions changed significantly with the measure of social isolation. The pandemic context imposed significant changes to the possibilities of establishing face-to-face interactions with other people, which can have repercussions as this condition continues. When investigating the perception of this situation and how people were being impacted in Brazil (in April, after a few weeks of social isolation during the pandemic), Bezerra et al. (2020) found that of the 16,440 participants, 39% revealed that not being able to live socially was being the greatest impact of social isolation. Certainly, this impact becomes more significant as time goes by.

Virtual tools and resources play an important role at this time, mediating the interaction between people and different contexts. Through virtual tools, exchanges between people focus mainly on verbal communication, through speech and writing. However, it is known that the perception of various non-verbal elements is equally important for the construction of interaction and connection with the other (Caspi et al. 2005). So it can be questioned whether this form of interaction provides engagement with the complexity and reciprocity necessary to meet the need to establish interpersonal relationships and favor development over time. Or even, it can be questioned whether, in these circumstances, interactions mediated by virtual resources can sufficiently replace face-to-face contact. These are some of the questions that require further research.

Regarding this theme, Guimarães (2009) has already argued that digital media, made available by social networks and cell phones, introduced new elements and changes in forms of socialization and subjectivity a decade ago. For the author, both the concept of belonging and the perception of place are resized based on the reorganization of social interactions mediated by internet tools. Extending this analysis approached by the author, Sant'Anna and Garcia (2011) discuss the changes promoted by the cell phone and point out other aspects that can be extended to virtual communications, such as changes in the perception of social roles previously defined by the presence or absence of people, changes in the distinction between public and private space, and the existence of simultaneity or juxtaposition of physical and virtual spaces. All of these changes bring transformations to the way we understand interactions. Guimarães (2009) adds that, at the same time that these tools bring new communicational elements that promote cultural transformations, the speed with which the virtual scenario and information are transmitted favors instability and transience, and these characteristics also mark the relationships when they overlap with face-to-face relationships. The author considers that, in this way, there is an “infinite postponement of presence” (p.20) that will have an impact on forms of interaction and identities.

The expression “postponement of presence” (Guimarães 2009) acquires another emphasis when we place it in the current context of the new coronavirus pandemic, in which social isolation is a necessity that brings change to access to different interaction contexts and that intensifies virtual communication. This modality of virtual interaction has assumed the role of maintaining the connection between people who have used these resources for an extended period of time during social isolation, raising questions about the impact that these interactions will have for children, youth, and adults over the time. Furthermore, if considering, for example, the concept of microsystem for bioecological theory, where proximal processes take place, the questions arise: Does the interaction mediated by the computer that promotes engagement over time have other properties? Does the establishment of proximal processes between people happen in this modality, without face-to-face interaction and or without the possibility of people to be in the same environment with physical characteristics? If so, in what context do the processes take place? It is not necessarily intended to answer these questions but to problematize the transformations and dilemmas that the moment raises.

On the other hand, by decreasing personal contact in other contexts, social isolation also promoted the intensification of coexistence in various domestic and family microsystems and changed the influences of the mesosystem—interrelation between different microsystems—in this context. With the use of technology and the internet, the family environment has been converted into a work environment, children’s studies, or a virtual meeting with friends, transforming routines and roles, which also change the configuration of proximal processes in this environment.

As Sheehy (1974) analyzes, crises such as those mobilized by the pandemic, in addition to interrupting the sequence and rhythm of the expected life-course, bring about upheavals in the roles and rules that comfortably defined the relationships established, requiring reorganization of routines, goals, interactions, and life projects. Like other (un)predictable events in life, the COVID-19 pandemic has disrupted older role chains. The pandemic context has turned many parents into teachers of math, science, geography, and homes in classrooms. Many elderly people, who until then had a very stable functionality, feeling totally independent and autonomous, suddenly became dependent on their children, grandchildren, or neighbors immobilized by the fear of going to the supermarket, pharmacy, or just taking out the trash. In this way, the pandemic has completely altered the people’s circulation path, which directly affects their social roles and forms of interaction.

In this sense, how does this new reality impact the body, perceptions, and construction of senses? To reflect on this question, we bring to the dialogue the framework of the enactive theory, particularly the theoretical aspect arising from the studies of Varela et al. (2017), which through the microscopic look at the interactional processes helps us to broaden the perspectives on the transformations experienced in this period.

### The Transformations of Corporeal Experiences in Interactive Processes

The enactive theory carries two central assumptions, which are particularly important for the topic addressed in this theoretical reflection. First is the idea that the way in which human beings know and think about the world (*cognize)* and construct meaning during their existence (*sense-making*) is through the body, its actions, and movements in its various dimensions. Thus, understanding human cognition as a bodily action means understanding that the subject as a cognitive system is embodied; its activity in the world in relation to others depends non-trivially on its body (De Jaegher and Di Paolo 2007). In other words, the subject’s experience depends on the possibilities of locomotion, perception, and engagement that its physical body is able to mediate in the interaction with the world, and these possibilities of locomotion, perception, and engagement are directly related to the individuals’ sensory-motor skills, their organic attributes, and the situation at the moment of interaction (Kyselo 2014). Therefore, the body is seen not only as a facilitator or an instrument for receiving stimuli but as a circumscriber, defining the individual’s experiences.

The second idea is that cognition is social and explained through the concept of intersubjectivity. Through the prism of the enactive theory, intersubjectivity is understood as mutual or participatory processes of construction of meaning conceptualized as *participatory sense-making*, which permeate perceiving, feeling, and thinking and are materialized in the actions with others during the interactions (De Jaegher et al. 2017). It is a relational process established with the world led by affection, guided by what is relevant to the individual, and supported by the individual’s biological organization—the physical body. An important element in this theory is that the interaction is not the sum of predefined regulatory processes between the subjects involved or, in other words, intentions are not necessarily previously and individually stipulated, but it is a process that has a life of its own and can be generated or transformed in the encounter through engagement. This engagement is constructed by the dialogues and explicit gestures, emphasizing that when we are interacting with another person there are non-verbal elements that circumscribe the communication process, which guide peoples’ attention and sensations and support their thoughts. These two fundamental ideas in enactive theory—that cognition is embodied and dependable on one’s actions and that cognition is social—places great emphasis on the two limitations imposed by the pandemic, the social interactions, and the possibility to engage (enact) in the world with joint activities with others. By bringing these two ideas to the discussion, we explicit how the social isolation and social distancing impact the body by constraining its actions, motions, and locomotion, and by doing so it creates constraints for how human beings *cognize* or understand the world.

To make this point clearer, empirical studies show, for example, that body posture influences people’s mood in social interactions (Osypiuk et al. 2018), facial muscles affect social judgments (Niedenthal et al. 2010), or that communication coordination of non-verbal and body synchronization are used to understand the action in collaborative tasks (Schneider and Pea 2014; Shockley et al. 2003). These and many other findings support arguments that cognition is completely dependent on the body in different cognitive processes that deal with co-specifics, such as the recognition and empathy of emotions, understanding of actions, joint attention, coordinated actions, and interactions in general (Reddy and Uithol 2015).

Under this perspective, it is important to consider two different situations: first, the absence or significant decrease of face-to-face social interactions during the pandemic; and second, the use of digital technology to mediate social interaction supporting social distancing during the pandemic. In the first situation, previous studies examining the effects of social isolation in different contexts have pointed out that the absence of physical contact and loneliness generated by the decreased social network can inhibit or decrease a series of physiological reactions that are directly linked to well-being and related to increased anxiety (Cacioppo et al. 2006) and the onset or worsening of depression (Cacioppo et al. 2010). Recent studies with data collected during the COVID-19 pandemic suggest that the compulsory social isolation, necessary during the pandemic in different countries, associated with other factors of vulnerability (e.g., feelings of fear, lack of resources and social support, restricted support network) can result in symptoms of post-traumatic stress, mental confusion, and anger, affecting not only the individual’s social behavior but also his metabolic functioning (Brooks et al. 2020).

The second situation consists of experiencing social distancing without the isolation or deprivation of sociability by using digital technology to support social interaction during the pandemic. In this way, social engagements can be identified by the complementarity of the speech content, synchronicity of the dialogue, regulation of movements and behavior, or even pairing of physiological stimuli. All of these elements are present during face-to-face interactions but are absent or are constituted differently in situations where interactions are mediated by other means than the body, such as in virtual interactions. What this means is that face-to-face contact or virtual resources define different perceptual possibilities that, consequently, will support specific engagements and actions. Thus, changing the configuration of social interactions during social isolation can affect the way the subject perceives the world and the ways in which interactions happen, reducing or significantly altering the provisions and affordances for the perception of the other and for the action with the other in diverse activities of life as we know it. Consequently, it can be considered that social isolation affects the subject’s physical presence in the world and the possibilities of movement, access, and contact with certain contexts, environments, objects, and people, impacting on the type of content that this subject experiences, feels, and makes sense.

### What are the New Communication Strategies During the Pandemic and How Does It Influence the Developmental Path?

During the pandemic, there has been an unprecedented increase on the use of technology and digital platforms supporting virtual interactions for educational, professional, and personal purposes, allowing people to carry on with routine activities supported by synchronous communication. However, as mentioned before, in such interactions, there is a transformation on how the engagements are carried out and how the actions of being together are performed.

Exploring the example of school contexts, the face-to-face learning environment, which supports a specific way of relating to teachers, peers, and the knowledge construction, is replaced by the virtual environment dependent on digital technologies and now experienced in the domestic space shared with the family. From the perspective of the bodily impacts, this change brings a new perceptual-motor field where interactions take place, and the new physical context impacts the way in which the students engage in school activities and interact with other people who are part of the process. Before the pandemic, the visual field during the learning situation encompassed an entire classroom, different materials, other people, and an environment built for the purpose of supporting learning; now, this field is reduced to a computer screen or tablet, indirect interactions with others, and an environment that is shared with other situations and social contexts. The new virtual learning environment demands less movement and more centralized attention and imposes a new body posture and a new type of engagement in the interaction, which involves a different pace for dialogue focused specifically on verbal communication. Without direct visual contact (mutual gaze) or any type of simultaneous action, this interactive situation creates a new perceptual reference to understand the interactive dynamic with the other colleagues. It also involves a linear rhythm in the conversation, since the platforms used for such interactions do not allow multiple dialogues at the same time.

All these changes in the perceptive-motor field impact perceiving and feeling and, consequently, individual’s thinking and acting requiring the development or improvement of specific interactive resources. One simple example to illustrate the reflection is that instead naturally relying on the mutual eye gaze and joint attention,^1^ teachers and students using online environments need to create other perceptual references to indicate that members of a group are paying attention to each other or to the same context and regulate behaviors during learning interactions (Huber and Helm 2020). These types of regulations guide teachers’ performance and decision-making during the lessons and are particular important for collaborative learning or even group discussions among the students.

Another important point is that from the enactive theory point of view, as cognition is not only embodied but it is also genuinely social, it consists of a constant dynamic that balances processes of distinction and participation that leads to the demarcation of an individual’s identities. Distinction can be understood as processes that help the subject to understand and experience his existence as an individual. Participations on the other hand are processes by which the subject allows being constituted by the other (Kyselo 2016). Together, distinction and participation establish the parameters, contours, and delimitations of the individual’s identities. The concept of identity in this theoretical approach is defined by the notion that biological (e.g., metabolism) and mental (e.g., thoughts and beliefs) phenomena are continuous and inseparable (see Di Paolo and Thompson 2017; Kyselo 2014), and their organization follows the same principle of autonomy and self-management described in autopoietic systems (Di Paolo et al. 2017). Such self-organizing processes can be found at various levels of the organism, such as in metabolic and neurobiological homeostasis or in sensorimotor integration, but they also occur beyond the individual organism at the social or collective level, and it is in that level that we are particularly interested. The relationship between subject and world is described in terms of nonlinear interactive dynamics, in which subject and environment are mutually built (Kyselo 2016). In this dynamic view, the subject’s identities are not previously established but continuously generated through this process of self-organization, in which the subject actively structures the exchanges with the environment. This process is called structural coupling and generates and maintains a form of systemic stability, which guarantees the maintenance of the individual’s identity(ies) to the same extent that it promotes constant expansion processes in the couplings.

Therefore, within the example of the school environment, the teacher-student and student-student relationships are, at the same time, a fundamental part of structural coupling in the school context and part of people’s identities. The bodily actions performed during learning situations (e.g., manipulation of objects, discussions with peers, movement in the classroom) shape the cognitive experience (Sullivan 2018) and take place within the perspective of maintaining the autonomy that regulates the identities created in relation to that specific context. These identities are co-constructed, they are co-dependent, and they only exist in relation to one another—one can only be a teacher if in the relation there is a student. Throughout the pandemic, the presence of the teacher, colleagues, and other professionals involved in the face-to-face learning process is replaced by the image, the voice, the idea of ​​what these subjects are doing, or even by other people such as the parents, which were not formerly part of that network of interactions. This replacement directly affects the configuration of interactions. Seeking to maintain this autonomous system, the forms of engagement are modified, and concepts, thoughts, and values ​​that were previously performed in a certain way and that caused the subjects to present certain actions will change during the pandemic. To keep their identities and the stability of the relationship during the interaction, members interacting must perform alternative actions, creating new strategies, for example, to assure intimacy, trust, and emotional connection pertinent to the relationship. The teacher might change her or his tone of voice, vocabulary, or even increase the number of times he or she verbally check how the students are doing to create connection with the students during the lesson; or it might develop a new way to give instructions that do not rely on juxtaposing verbal explanation and gestures. The point made here is that because of the new configuration of the interaction, how it changes the engagements and actions, and the need to maintain the individual identities, a new set of skills develops.

Additionally, it is important to consider the long-term impacts on the construction of meaning about the given reality. In the context of the pandemic, the meanings of teaching and learning are challenged and redefined because the circumstances of social isolation have broken the borders between relations that before the pandemic were clearly defined, such as the role of parents in children’s learning processes. One good example is how Chinese parents are changing their beliefs and attitudes around young children’s online learning during lockdown of the COVID-19 pandemic. Before the pandemic, the use of technology and digital learning environments by the schools was a sign of pedagogical advancement. However, when the technology is used as a substitutive learning environment, parents are resisting and tending to reject the new modes of teaching and learning (Dong et al. 2020). The rejection is based on observations of children’s inadequate self-regulation, shortcomings of online learning, and the acknowledgment on parents’ own lack of time and professional competences in supporting children’s learning. In this case, the reconfiguration of the interaction brought parents physically closer to the learning process, demanding active engagement and a specific set of action that have challenged the references of what learning and teaching means, as well as impacted individuals’ identities within the relations established among teachers, parents, and students.

These transformations would be irrelevant if the time of exposure to this way of interacting was short and the usage of the technology would not have been significantly increased. Even though digital resources were already known tools, their extensive use in different types of interaction is a new phenomenon. Therefore, it is precisely in the combination of time and context that the developmental questions raise. If the “careful orchestration of where and when a child is asked to remember something, or repeat an activity, may greatly facilitate his or her performance” (Reed 1996, p. 147) is considered, it is also necessary to ponder that this different way of engaging in the world will influence how children are learning and developing. Thus, it is not just a question of limiting face-to-face social contact, restricting the spaces where collective actions materialize. In fact, we are in a process that transforms the way we perceive, feel, think, and consequently, act in the world and exist as social beings.

Therefore, dialogue with the enactive approach allows the discussion on development based on the potential contained in the dynamics of interaction and significant engagements—in intersubjectivity (Reddy 2008). From this perspective, it can be said that the development will occur when, in the interactive dynamic, the maintenance of the subject’s identity (identities) and the expansion of the subject’s actions in the world (expansion of his movements and actions) coexist.

Reflecting on the question of *whether the social isolation adopted as a measure to combat the COVID-19 pandemic also represents a crisis of interpersonal contact*, we understand that the most objective answer would be yes. The pandemic imposes a stress-generating movement in interpersonal relationships that changes the status of the individuals, interfering from the most concrete level of their existence—their corporeality, to the most complex social systems. However, it is by thinking about the question on *how the pandemic brings new circumscribers to our interactive constitutive experiences* that we find crucial elements to move the discussion forward. In the sphere of interpersonal relationships, the pandemic accentuated, or highlighted the essentiality of human activity, its sociability, and through the challenges imposed in maintaining affective ties or in the (re)signification of relationships, it revealed that there are many aspects of this interpersonal interaction that still have not been explored in human experiences or in the sciences that study them.

## Final Considerations

Throughout this theoretical study, we sought to reflect on transformative and constitutive processes mobilized by the current socio-historical context of the pandemic caused by the new coronavirus (SARS-CoV-2). As an axis of reflections, it was considered that preventive measures to contain the spread of the virus imposed several reorganizations and social restrictions that redirected people’s routine, delimited and remodeled their bodily experiences with other people, with different contexts, public spaces, and specific social situations, such as interactions at the school and family context, circumscribing important worldwide transformations at an individual and social level. Faced with this scenario, approaching the topic from the perspective of crises and their transformations in developmental processes, they were discussed as the vulnerabilities and opportunities that characterize historical events of this nature—(un)predictable, which mark a period of upheaval, critical transition (or “crisis”)—can become a turning point in life courses, mobilizing adaptive processes and with repercussions on human development processes.

From the perspective of the changes promoted by the pandemic context, it is observed how human development does not occur in an isolated way in the organism, restricted to intra-individual processes, but in the bi-directionality of the organism-environment. The situated and relational character of human development stands out, through the understanding that human development includes not only a single being but a set of people in interaction processes established in different contexts (Rossetti-Ferreira et al. 2007). Thus, in the present theoretical study, it was emphasized that the developing person is part of a network of relationships and, as stated by Rossetti-Ferreira et al. (2007), the analysis of development processes must consider the person as part of systems and must seek to grasp the relationships between this person and the phenomena of his surroundings. By transforming ecological contexts, the pandemic also transforms people and their repertoires of life. Multiple development trajectories are possible; the paths can become unexpected and occur in a “continuous, constant flow, co-building and transforming while contributing to constitute the other and the situation” (Rossetti-Ferreira et al. 2007, p.30).

To deepen the analysis of these change processes, the focus was on transformations and new arrangements in interpersonal interactions during the pandemic, including a reflection on the intensification of the mediation through digital technology and communication tools. We understand that the enactive approach brought important and complementary elements to the reflection exercise proposed in this article, enabling the understanding of how the transformations promoted by crises are articulated with our ways of perceiving, feeling, thinking, and interacting when establishing interactions with the world that is important and meaningful to us.

It is considered that, with regard to interpersonal interaction processes, the pandemic evidenced the value of human relations for maintaining the organicity of social systems. The repositioning of relational contexts, the body and its experiences, and the impossibility of exercising sociability in the way we understood and performed it force us to adjust the mechanisms of engagement so that we can maintain our identities and connect with other people. Thus, it is precisely in this adjustment process that we can expand our action repertoires, which is understood here as a developmental process, (re)signify the world of which we are a part and which we build, in addition to redirecting our actions that, consequently, constitute the social spaces and practices around us.

Based on what was discussed, one of the attributes of the crises that deserves to be highlighted refers to the opportunities for change, leading individuals and societies to reflect on the need to invest in viable, sustainable alternatives for different people, social groups, and communities, enabling the development of coping strategies and personal and community resilience in a comprehensive manner. Thus, it can be considered that the pandemic and its repercussions also revealed that it is in the midst of strengthened social interactions, marked by mutual engagement and support, building support networks and relationships, belonging and affective ties, that the development of human potentialities is promoted, finding fertile ground for individual and collective transformations (Juliano and Yunes 2014).

Regarding the limitations of the reflection presented here, it is important to mention that the additional impacts arising from economic and social limitations, such as, for example, the access to virtual technologies that enabled the continuity of some processes, such as schooling, were not addressed. Access to virtual resources (e.g., internet, smartphones, tablets, computers, software) is not universal, and there is an inequality in the possibility of establishing or maintaining interpersonal relationships through technology. Cultural specificities that significantly impact the experience of social isolation or compliance with sanitary restrictions were also not addressed. Although they are quite relevant dimensions, in this work we chose to address other aspects. Thus, it is important to carry out other reflections and investigations, such as the impact of the pandemic and social isolation among different social groups.

## Footnotes

1 For the definition of joint attention, see Moore and Dunham (1995).
